# Love thy neighbour: Feral buffalos show greater space use, resource overlap and encounters during the wet season in the Northern Territory

**DOI:** 10.1002/ece3.70345

**Published:** 2024-10-22

**Authors:** Kyana N. Pike, Justin Perry, Eric Vanderduys, John P. Y. Arnould, Andrew Hoskins

**Affiliations:** ^1^ Health & Biosecurity Environment Commonwealth Scientific and Industrial Research Organisation (CSIRO) Townsville Queensland Australia; ^2^ Northern Australian Indigenous Land and Sea Management Alliance Brinkin Northwest Territories Australia; ^3^ Environment Commonwealth Scientific and Industrial Research Organisation (CSIRO) Brisbane Queensland Australia; ^4^ School of Life & Environmental Sciences Deakin University Melbourne Victoria Australia; ^5^ College of Public Health Medical and Veterinary Sciences, Australian Institute of Tropical Health and Medicine James Cook University Townsville Queensland Australia

**Keywords:** Asian water buffalo, biosecurity, continuous time movement models, decision support, disease risk, feral animal management, seasonal space use

## Abstract

Managing feral water buffalo in the Northern Territory is a formidable challenge. As an introduced species, buffalo are associated with a myriad of biosecurity, economic, cultural and environmental issues ranging from overgrazing, decreased water quality, disease vectors to the destruction of cultural assets. Nevertheless, the buffalo are also a harvestable resource that can support economic development of the region. To mitigate some of the biosecurity, economic, cultural and environmental risks they pose and manage buffalo effectively, we need a detailed understanding of their spatial and behavioural ecology. However, several factors make understanding how best to manage the dense populations of wild individuals challenging as buffalo inhabit remote areas with limited infrastructure and accessibility and their large size and often aggressive nature can make them difficult to observe in otherwise inaccessible areas. GPS tracking allows for high‐frequency data collection and surveillance of individual buffalo. Here, we investigated how the different seasonal periods of a Northern Territory floodplain area shaped patterns of habitat use for 17 buffalo tracked over 16 months. We found in the dry season, buffalo space use is restricted, and the size of home ranges are significantly smaller than in the wet season. During the wet season, buffalo expand their home range area as well as their social encounter area with other buffalo. These differences in their space use and social patterns suggest that increased disease surveillance may be needed for the wet season when buffalo are more likely to share space and interact. During the dry season, however, buffalo movement is more predictable and restricted, suggesting greater optimisation opportunities for buffalo management. Results from these models can be used by land holders, Traditional Owners and wildlife managers to make evidence‐based decisions to improve buffalo management with respect to disease risk, sustainable harvest and damage to environmental and cultural assets.

## Graphical abstract

Feral buffalo in the Northern Territory are associated with several cultural, environmental and biosecurity challenges, and to mitigate these issues, we GPS tracked 17 buffalo over the wet and dry season. We found in the dry season that buffalo space use is restricted, their movement is more predictable, there is less social interaction and the size of their home range is significantly smaller than in the wet season. Results from these models can be used by land holders, Traditional Owners and wildlife managers to make evidence‐based decisions to optimise buffalo management with respect to disease risk, sustainable harvest and damage to environmental and cultural assets.

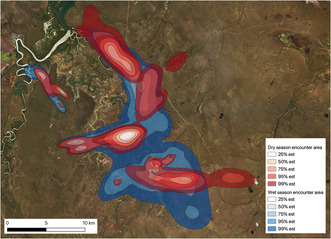

## 1 INTRODUCTION

Throughout their Australian distribution, feral swamp buffalo (*Bubalus bubalis* Lydekker) are associated with a multitude of biosecurity, economic, environmental and cultural issues (Collier et al., 2011; Robinson & Whitehead, 2003; Skeat et al., 1996). The feral populations are restricted to the Northern Territory (NT), but there are a few records from north Queensland, of unknown status (Bowman & Corbett, 2023). The number of buffalo has fluctuated over decades due to periods of commercial hunting, then unprecedented growth and then deliberate culling (Werner, 2014), but, today, numbers again have risen to very high levels and continue to grow.

Small numbers of swamp buffalo were originally introduced to the NT in the 1820s and 1840s from Southeast Asia, to start a buffalo industry (McMahon et al., 2013; Skeat et al., 1996). This failed, but a feral population became established and spread across the north of Australia (the ‘Top End’). Through the first half of the twentieth century, the numbers were generally held in check by hunting, especially for their hides that were exported as valuable sources of industrial belting. During WWII, the export was greatly curtailed simultaneously with the invention of artificial rubber, and commercial hunting of the buffalo ceased. Numbers increased greatly from 1960 to 1980 (Petty et al., 2007; and references therein). In fact, by the 1970s, buffalo populations in the NT had reached over 340,000. A high prevalence of tuberculosis (*Mycobacterium bovis*) infections prompted the Australian Government to introduce a large‐scale $850 million eradication programme (the Brucellosis and Tuberculosis Eradication Campaign: BTEC) during the 1980s to cull feral cattle and buffalo from pastoral lands to curb risks of tuberculosis and brucellosis threatening the livestock industry (Bradshaw et al., 2012; Freeland & Boulton, 1990). Under the BTEC programme, the numbers of feral buffalo were dramatically reduced across the Top End with some local densities (e.g. northern Kakadu National Park) originally as high as 34 buffalo per km^2^ dropping to less than 1 per km^2^ by the 1990s (Davies et al., 2023; Ridpath et al., 1983). We note that the BTEC programme was not conducted in Arnhem land, and buffalo later moved westwards back into Kakadu National Park.

Overall, subsequent culling and management efforts throughout the region have not been enough to suppress the buffalo populations (Campbell et al., 2021). Buffalo are well adapted to riverine and wetland areas that make up much of the northern coastal zones of the NT, so populations can reach high densities. Population size and mortality are modulated by seasons (Freeland & Boulton, 1990; Tulloch & Grassia, 1981). Buffalo form family clans of 10–30 individuals (Tulloch, 1979) which then can aggregate to form herds of up to 500 individuals in a local area (Jesser et al., 2008). Their high densities, coupled with social aggregations, mean that buffalo continue to be a biosecurity risk in need of agile surveillance and monitoring that can be assisted by considering the social dynamics of buffalo in these remote areas (Bradshaw et al., 2012).

Individual buffalo exhibit high site fidelity within their home ranges but also move across the landscape daily and these movements vary with season (Campbell et al., 2021; Forrest et al., 2024; Tulloch, 1969). Rainfall is monsoonal and highly seasonal, with 90% falling between November and March (Taylor & Tulloch, 1985), and from observational studies, we know that buffalo behave differently in the wet and dry seasons. In the wet season and for several months thereafter, buffalo swim onto the inundated floodplains and feed on vegetation there, then return to the mesic fringing woodlands in the evenings to rest overnight, where mating occurs and where females tend to their calves. In the middle to end of the dry season, the floodplains are devoid of vegetation and open water is found only in some small ‘puddles’ so much time is spent wallowing in mud to remain cool; little food is eaten and most sparse grazing is done on the fringing woodlands. The buffalo movements, feeding and wallowing create many problems in the extensive seasonal wetland areas (Campbell et al., 2021; Petty et al., 2007; Taylor & Friend, 1984; Tulloch & Litchfield, 1981; Williams & Ridpath, 1982). For example, repeated wallowing and trampling close to the river courses can lead to saltwater intrusions onto otherwise freshwater areas, soils become compacted and high sedimentation loads decrease the water quality of many wetlands, billabongs, springs and rivers (Petty et al., 2007; Taylor & Friend, 1984; Tulloch & Litchfield, 1981 and references therein). Through their overgrazing and selective herbivory, buffalo are also responsible for changing vegetation communities, consuming many native plants and promoting the spread of invasive vegetation (Mihailou & Massaro, 2021; Petty et al., 2007; Petty & Werner, 2010; Werner et al., 2006). Furthermore, buffalo and fire have been demonstrated to interact and alter demographics of vegetation communities (Werner, 2005; Werner et al., 2006). These combined direct and indirect changes to the environment have flow‐on effects that also decrease the abundance, distribution and recruitment of several native vertebrates such as magpie geese, dusky rats and other small vertebrates (Skeat et al., 1996).

A large proportion of the NT (48% of the land mass and 80% of the coastline) is owned and managed by Indigenous Australians who have strong connections to traditional culture, practices and land management (Austin & Garnett, 2011). The long history of buffalo in the NT has had mixed impacts for the social and cultural landscape (Collier et al., 2011; Robinson & Whitehead, 2003; Russell, Ens, & Rangers, 2020a). On the one hand, large numbers of buffalo can cause significant damage to cultural assets and practices, both directly such as damaging sacred Indigenous rock art, and important gathering sites and increasing health risks through water‐ and soil‐borne disease (Ens et al., 2010; Robinson et al., 2005; Russell, Power, & Ens, 2020b), and indirectly such as reducing native vertebrate fauna populations for hunting such as magpie geese (Ens et al., 2010; Skeat et al., 1996). Additional negative cultural impacts are also implicated in the environmental degradation that buffalo are responsible for, as Traditional Owners and Indigenous land managers have strong cultural ties to the land and its health (Robinson et al., 2005; Russell, Ens, & Rangers, 2020a). Alongside the negative cultural and environmental factors, however, are positive attitudes towards buffalo as they can offer benefits to local land holders as a valuable resource (Bowman & Robinson, 2002; Collier et al., 2011; Ens et al., 2010; Robinson et al., 2005). The buffalo mustering industry brings with it economic and employment opportunities and Indigenous‐led wildlife enterprises through the harvest and sale of buffalo and/or buffalo products (e.g. hides) for domestic and international export markets (Austin & Garnett, 2011; Collier et al., 2011). Additionally, buffalo are frequently hunted by Aboriginal people as an important food source for many Indigenous communities, especially when other food is not available for large gatherings or ceremonies (Bowman & Robinson, 2002; Ens et al., 2010; Robinson et al., 2005). In sum, managing feral buffalo on Indigenous land requires flexible strategies that can accommodate multiple approaches and goals such as preserving populations for harvest in some areas, while suppressing them in others.

To adequately manage and mitigate these issues. Traditional Owners, environmental managers and landowners thus need detailed information on buffalo ecology that relates to how buffalo move through the landscape, where to locate them and their intraspecific dynamics. However, due to the remoteness of buffalo inhabited areas, there is limited vehicle access and infrastructure, and the large size and often aggressive nature of individual buffalo make them difficult to observe, measure and manage in the wild (McMahon et al., 2010). Early studies often used aerial mechanisms, locating and counting buffalo from the air (e.g. Ridpath et al., 1983) or else observational studies of buffalo herds in the field (e.g. Tulloch, 1969, 1979; Tulloch & Cellier, 1986). Previous research has contributed greatly to our understanding of buffalo movement, behaviour and social structure, particularly for groups of animals, but detailed longitudinal data on individual animals are harder to come by (but see Forrest et al., 2024; Campbell et al., 2021). More recently however, advances in Global Positioning System (GPS) tracking technology have allowed managers to safely collect high‐frequency, remotely sensed data on animal locations in time and space that have overcome some of the issues of working in logistically challenging locations.

The present research is part of a larger project that broadly seeks to support the administration and management of free‐ranging buffalo on Indigenous lands. Our aims here were to provide valuable ecological insights into the seasonal patterns of movement, space use and social dynamics of feral buffalo to facilitate management activities relating to the protection of environmental and cultural values, development of economic opportunities and assessment of the impact of feral animal activities. We compared seasonal home range area, home range overlap and encounter area of 17 female feral buffalo GPS tracked in the Djelk Indigenous Protected Area (IPA) in a remote floodplain over a 16‐month period. Results from this study can be used by land holders, Traditional Owners, and wildlife managers to make evidence‐based decisions that optimise buffalo management with respect to various issues such as disease risk, sustainable harvest and damage to environmental and cultural assets.

## 2 MATERIALS/METHODS

### 2.1 Study site

**FIGURE 1:**
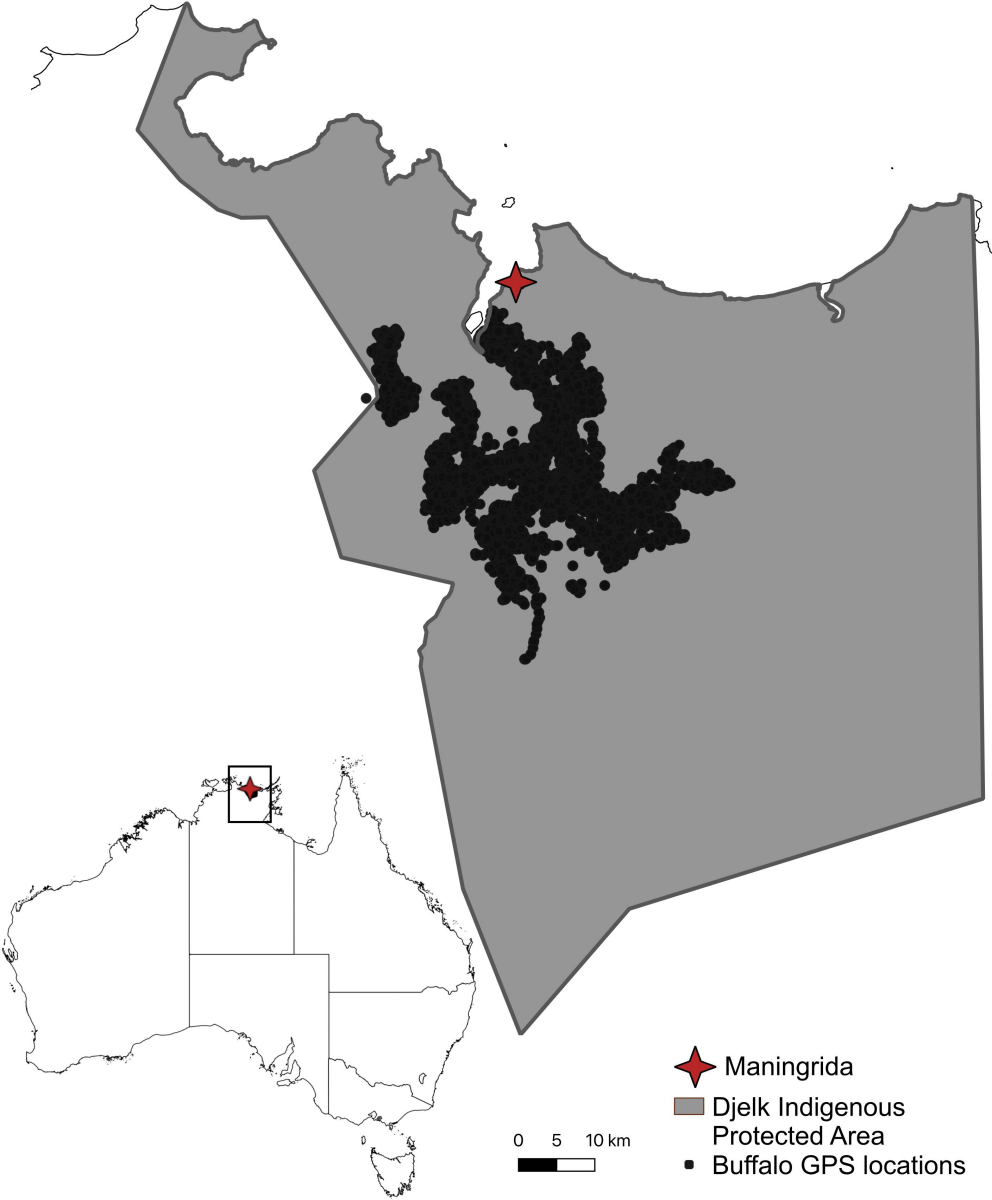
Overview of GPS locations of 17 female buffalo recorded over the study period within the Djelk IPA. The buffalo locations are mostly dispersed over the Tomkinson River floodplains. The location of the Djelk IPA and the Indigenous township of Maningrida in Northern Territory, Australia, is provided in the black box in the inset map.

This study took place in the Djelk IPA, located in the NT of Australia (see Figure 1). The Djelk IPA was established in 2009 and covers approximately 670,000 hectares of land and sea country that is under the management of the Traditional Owners for its sustainable use, and protection of biodiversity and cultural assets. The region has a tropical climate with an annual monsoonal wet season (typically November to April), followed by months of drought and wildfires during the dry season (typically May to October). The study area was the Tomkinson River floodplains and surrounding low hills, centred around 30 km south of the town Maningrida, This town is also one of the largest remote Aboriginal communities in the NT with more than 2500 residents and 30 outstations or homelands (West Arnhem Regional Council, 2024). This region is dominated by extensive seasonal floodplains and wetlands dominated by grasses and sedges and with fringing paperbark (*Melaleuca* spp.) woodlands. The slopes of the low hills are covered with tall open woodlands dominated by *Eucalyptus miniata* A. Cunn. Ex Schauer and the highest ground by Darwin stringybark (*Eucalyptus tetrodonta* F. Muell). Monsoon rainforest are found in fire‐protected areas.

### 2.2 Buffalo tagging and tracking

In collaboration with Djelk rangers, buffalo chosen for tracking were first located by helicopter and immobilised by tranquiliser darts following the methods of (McMahon & Bradshaw, 2008). Males tend to be heavier than females (Australian Freedom from Hunger Campaign, 1977), therefore requiring a heavier dart and a higher tranquilliser dose. This fact would mean a shorter firing range and increased risks with the helicopter and anaesthetic dart combination we used. For this reason, we targeted only adult females over 3 years of age for our study. Seventeen females were darted tranquilliser and then fitted with collars with very high‐frequency‐enabled custom GPS (Telonics MOD‐500). All animal handling procedures were performed by suitably qualified personnel trained in the appropriate capture and handling techniques under the approval of CSIRO Wildlife and Large Animal Ethics Committee (permit number 2017‐27). Each GPS collar was configured to collect an animal's location (±10 m accuracy) once per hour for 15 months, from 25th July 2018 until 31st October 2019, which essentially covered a dry season, then a wet season and then a second dry season.

### 2.3 Home range estimates

We determined robust estimates of seasonal home range size using the ‘ctmm’ package (Calabrese et al., 2016) in R v.4.0.2 (R Core Team, 2020). We first identified and removed outliers (e.g. if the animal was located in the ocean) using the ‘outlie’ function where buffalo locations were unrealistic based on their speed or distance (Calabrese et al., 2016). Over the tracking period, we experienced some tag failure so that not all individuals were continuously tracked for the entire period (mean number of locations per buffalo = 7445 ± standard error 710, range = 218–10,417). Then, we separated the tracking dataset into wet season and dry season periods to give an estimate of space use for each individual in three seasonal periods (i.e. dry season, wet season and dry season). Specifically, the three seasonal periods of tracking data and the number of buffalo used during each season were as follows: to November 2018 dry season (*N* = 17 buffalo), November 2018 to April 2019 wet season (*N* = 16 buffalo) and April to October dry season 2019 (*N* = 11 buffalo).

To create each seasonal home range, we first estimated the underlying movement models for each buffalo and used that to fit autocorrelated kernel density estimates (AKDE) to extract the 95% home range estimates fit with 95% confidence intervals for the estimate (mean effective sample size (Ne) for area = 120, range 9–360). We then tested for significant differences for home range size between the wet and dry seasons with a linear mixed‐effect model, with tag id as a random effect using the ‘lme4’ package (Bates et al., 2015).

### 2.4 Home range overlap estimates

To develop a better picture of the social dynamics of space use and how this differs between the wet and dry seasons, we also estimated the level of home range overlap between individuals using the overlap function of the ctmm package. The overlap function takes the 95% AKDE home range estimate and uses Bhattacharyya coefficients (Winner et al., 2018) to determine the level of overlap between the estimated utilisation distributions of different individuals, with higher values indicating greater home range overlap (Winner et al., 2018).

To better understand site fidelity, and the stability of home ranges over time, we compared the overlap of buffalo home ranges for the same individuals for which we had three seasonal periods (*N* = 11).

To investigate shared resource use and to complement our investigation of the seasonal social dynamics, we also estimated home range overlap among different individuals within each season (*N* = 17 for 2018 dry season, *N* = 16 for 2018–2019 wet season and *N* = 11 for the 2019 dry season).

### 2.5 Social encounter estimates

To better understand the social dynamics of buffalo space use both within and between seasons, we also estimated the utilisation distribution of encounter events among individual buffalo for our three seasonal periods. The conditions for a local encounter event can be identified in the tracking data, when two individuals occur at the same place at the same time (Noonan et al., 2021). This information from any two animals' space use can then be used to estimate the probabilistic distribution of encounter locations (Noonan et al., 2021). The encounter function of the ctmm package uses the 95% ADKE home range estimate of individuals to estimate the spatial distribution of encounter locations (termed conditional distribution of encounters or CDE) with confidence intervals generated from the home range estimate. This allows us to determine the distribution of encounter space among buffalo and identify areas where there is a higher probability of social encounters among buffalo within each of these seasonal periods.

## 3 RESULTS

### 3.1 Seasonal home ranges

**FIGURE 2:**
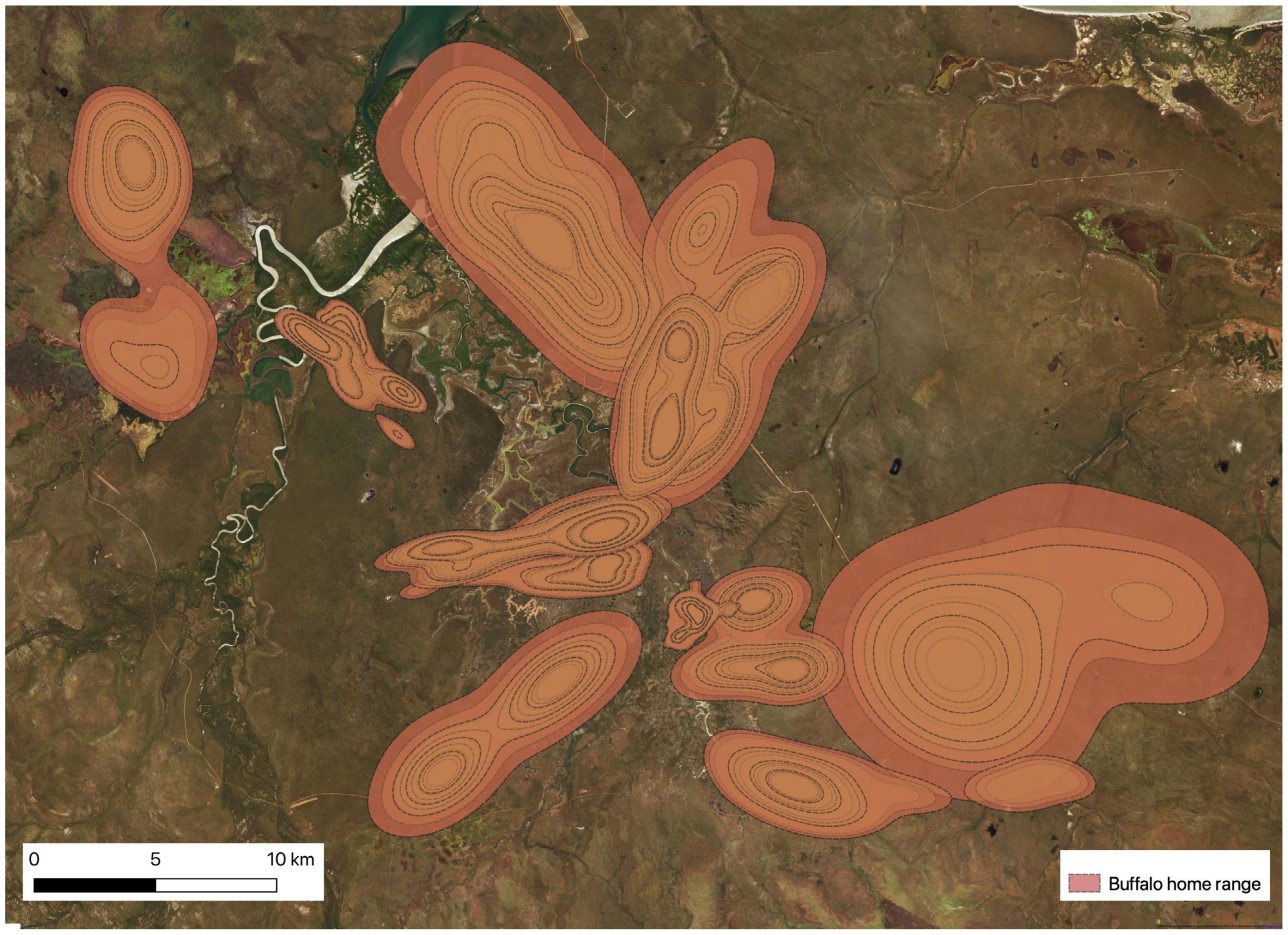
Dry season home range estimates during 2018 for 17 buffalo in the Djelk IPA in Northern Territory, Australia. Figure shows the 25%, 50%, 75% and 95% isopleths as dashed lines for each buffalo home range. During the dry season, home ranges tended to be contracted around the diminishing sources of water and vegetation.

**FIGURE 3:**
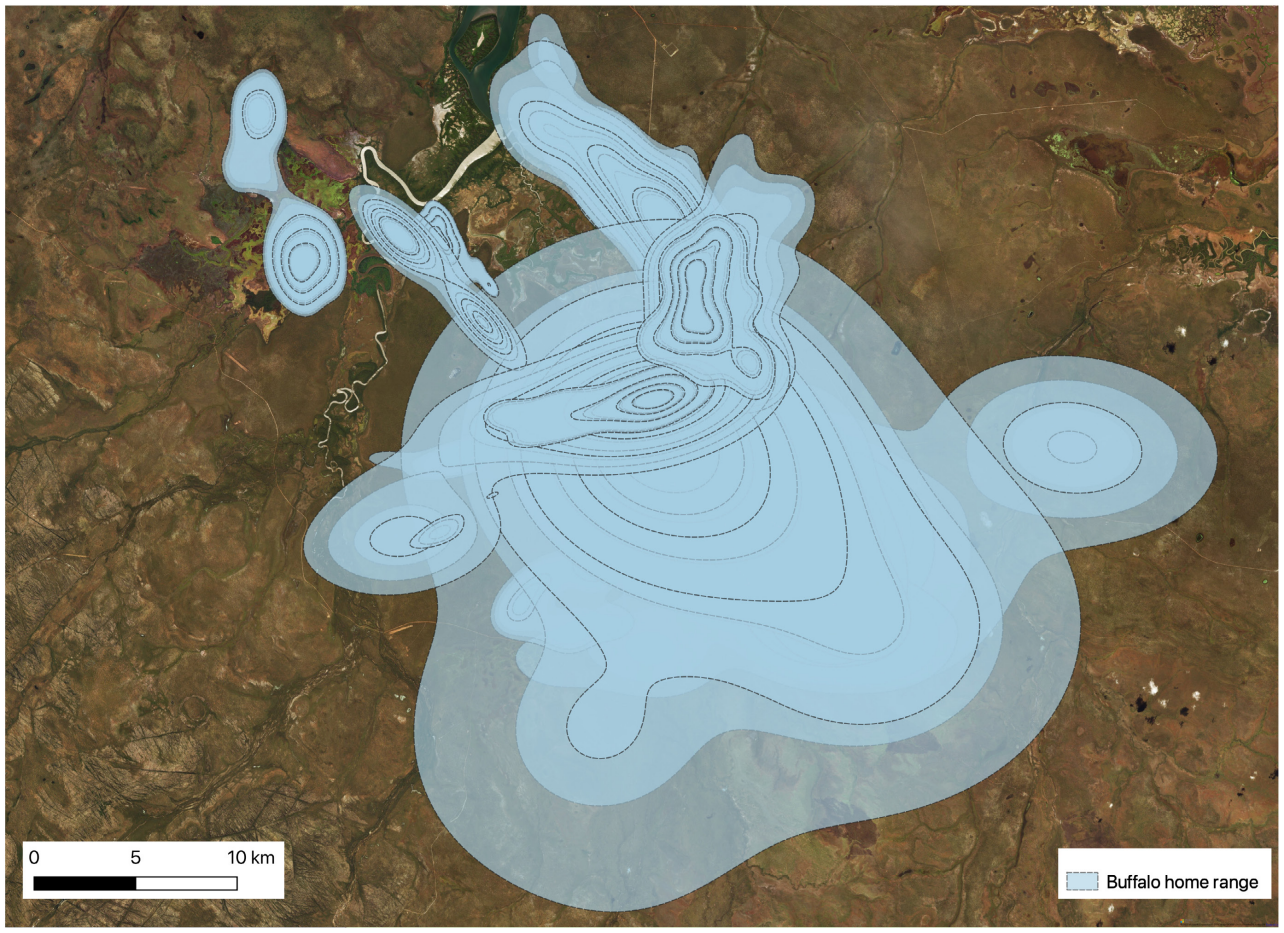
Wet season home range estimates for 16 buffalo in the Djelk IPA. Figure shows the 25%, 50%, 75% and 95% isopleths as dashed lines for each buffalo home range. During the wet season, home ranges are larger and tend to expand into the floodplain where both water and vegetation is more abundant than during the dry season.

During the dry seasons, the combined average home range size was 34.48 km^2^ (95% CI 6.10–62.34 km^2^; Figure 2; see Figure 2 an example of the 2018 dry season). The average wet season home range was 84.16 km^2^ (95% CI 5.183–93.458 km^2^; Figure 3). This was significantly (*t* (29) = 2.24, *p* = .025) larger than average home range size in the dry season average. See Tables S1–S3 for full tables of home range area by individual buffalo.

Range residency behaviour was strong within each season. The average home range crossing time was short for both seasons, but did differ between them. For the dry season, it was 1.93 ± 0.48 days and for the wet season, 3.19 ± 1.08 days. In contrast, estimates of their average speed was very similar between seasons (14.05 km per day for the dry season and 14.26 km per day for the wet season).

Female buffalo also showed high fidelity to their individual home ranges (mean proportion of home range overlap = 0.731 ± 0.037, range = 0.013–0.943), and tended to reuse the same areas between seasons. See Table S4 for a full table of home range overlap for the individual buffalo among seasons.

### 3.2 Social encounter estimates and home range overlap

**FIGURE 4:**
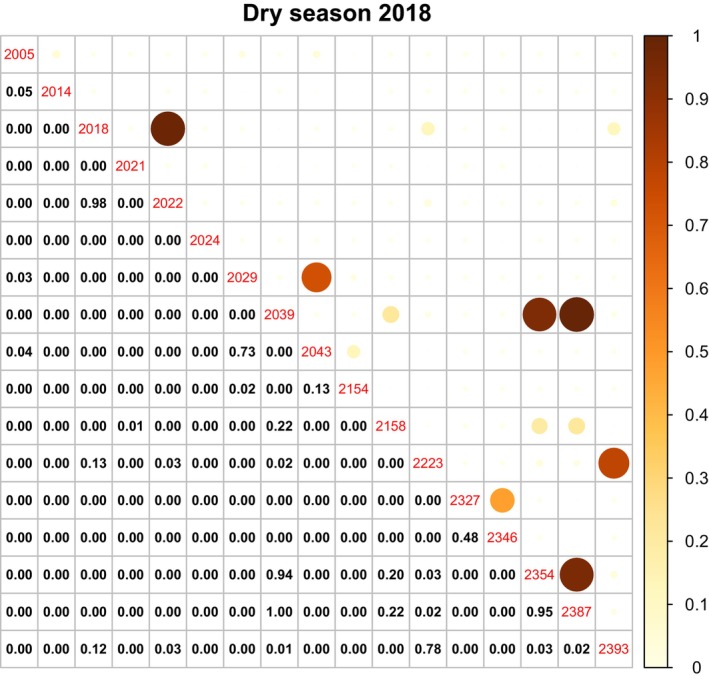
Home range overlap in the 2018 dry season, showing pairwise comparisons among all tagged buffalo in the Djelk IPA. The tag ID of the 17 buffalo is shown as red central numbers. The lower panel has the numerical proportion of home range overlap between buffalo pairs. The upper panels depict the overlap values visually, with the larger and darker circles indicating greater home range overlap.

**FIGURE 5:**
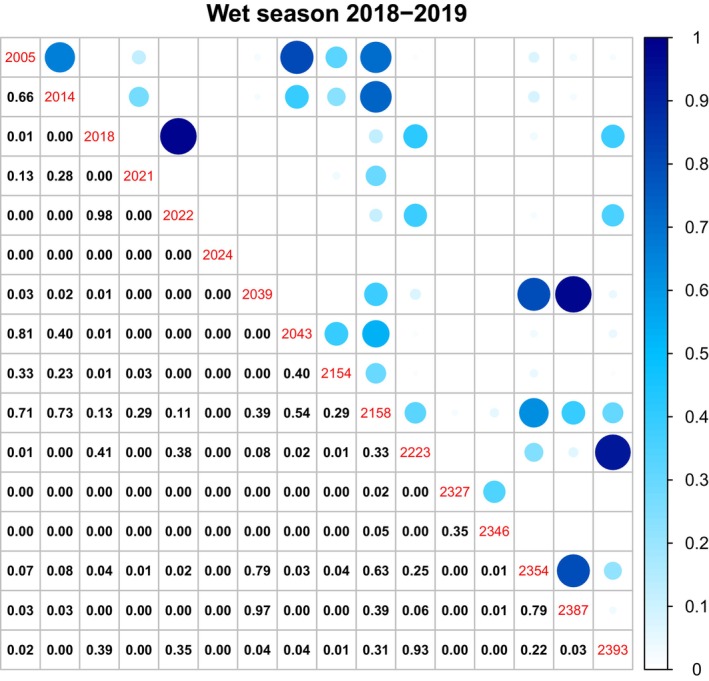
Pairwise comparisons of home range overlap in the Wet Season, showing pairwise comparisons among all tagged buffalo in the Djelk IPA. The tag ID of the 16 buffalo is shown as red central numbers. The lower panel has the numerical proportion of home range overlap between buffalo pairs. The upper panels depict the overlap values visually, with the larger and darker the circles indicating greater home range overlap.

We found a greater partitioning of space during the dry season, compared to the wet season. Over the dry season, buffalo generally had lower home range overlap with other buffalo (mean proportion of overlap = 0.058 ± 0.015) compared to the wet season (mean proportion of overlap = 0.132 ± 0.022). Furthermore, during the dry season, buffalo also shared their habitat with other buffalo less often as only 10% of different buffalo pairs shared more than 5% of their home range area, compared to 30% of buffalo pairs in the wet season (Figures 4 and 5 and see Figure S1 in the supplementary material document).

Overall, most buffalo avoided sharing space with other buffalo. However, some buffalo consistently had very high proportions of overlap (approximately 5% of buffalo pairs had a home range overlap of 70% or more) and a few other buffalo were persistent through all the seasons (Figures 4 and 5; also see Figure S1).

### 3.3 Encounter space

**FIGURE 6:**
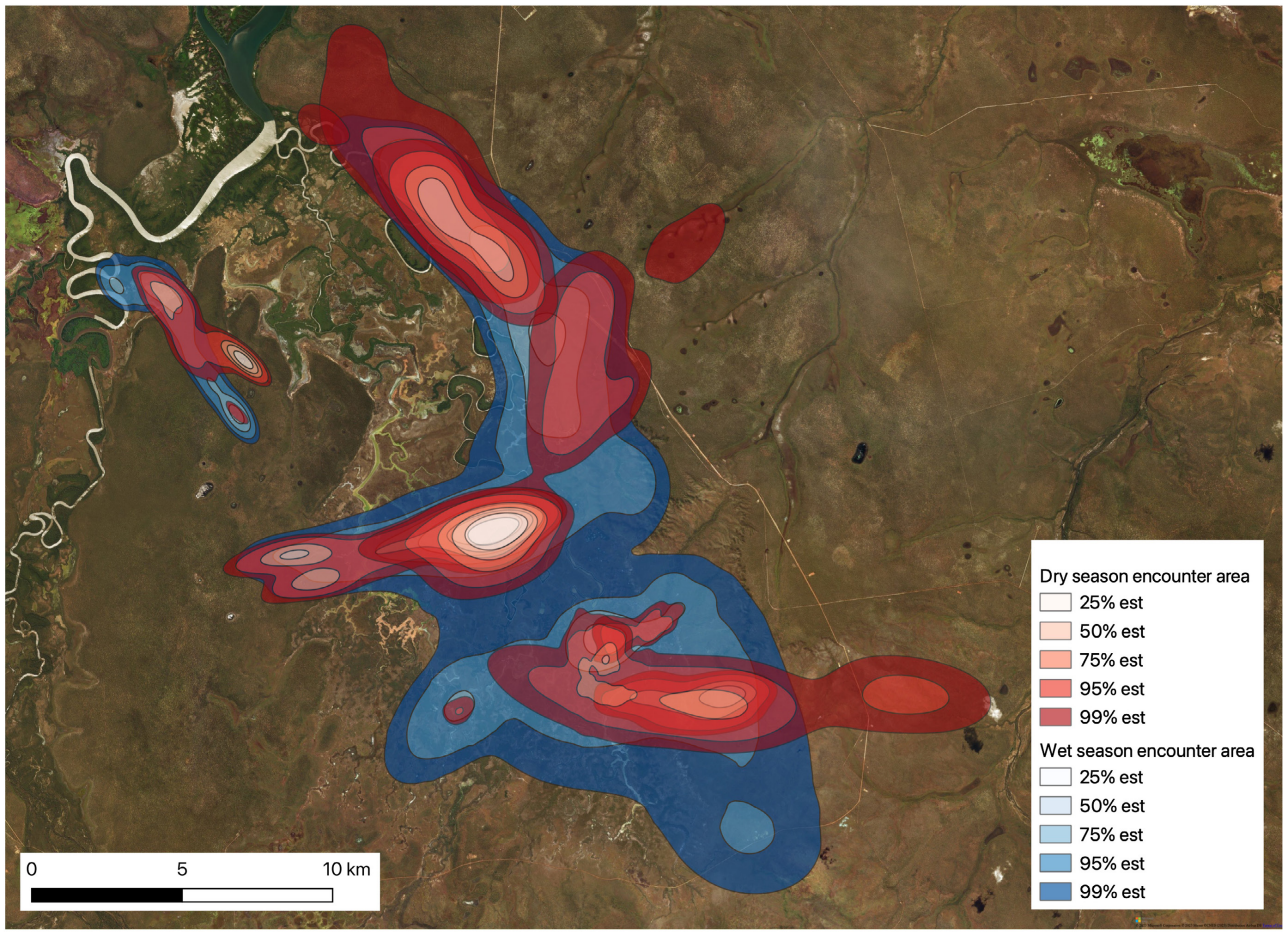
Comparison of the encounter areas during the wet and dry (combined 2 years) seasons for all tagged buffalo in the Djelk IPA. Buffalo tended to have a larger encounter area during the wet season (shown in blue) compared to the dry season (shown in red). The conditional encounter estimates show the 25%, 50%, 75%, 95% and 99% isopleths for both seasons. The lighter the area, the greater the density of encounters.

The encounter space in the wet season was more than twice the extent of the encounter space in the dry season (Figure 6). We estimated the extent of the potential encounter space (from the 99% isopleths for encounter area). For the 2018 dry season, encounter space was 96.01 km^2^ (95% CI 89.13–103.14) and in the 2019 dry season, it was 70.90 km^2^ (95% CI 58.90–83.99), compared to that in the season where encounter space was 203.18 km^2^ (95% CI 193.95–212.62 km^2^). Overall, neither the 2018 nor the 2019 dry season produced encounter space greater than the average encounter space of the wet season.

## 4 DISCUSSION

Through our high‐resolution tracking study of individual buffalo, we have generated novel insights into the movement, space use and social dynamics of feral buffalo that supports two decades of foundational work of observational field studies. Here, we found strong seasonal differences in the patterns of space use, resource sharing and social dynamics among individual buffalo in the floodplain area of the Djelk IPA of the NT. Our results showed that buffalo home range size fluctuates with season, with wet season home ranges expanding out to encompass a larger area, and then contracting in the dry season. This result likely reflects the buffalo altering their space use in relation to the availability of forage, water and thermoregulatory resources (as either shade or water), which are some of their essential habitat requirements (Tulloch & Cellier, 1986; Tulloch & Litchfield, 1981) as well as reproductive activities and presence of calves (Tulloch, 1979; Tulloch & Grassia, 1981; Werner, 2014).

During the dry season, wetlands contract and buffalo become restricted to areas where water, shade and forage remain available (Williams & Ridpath, 1982). Water buffalo are reliant on free standing water and an adult drinks approximately 21 L a day (Williams & Ridpath, 1982). For cooling, buffalo wallow in wet and muddy areas, or seek shade during hot periods (Forrest et al., 2024; Tulloch & Litchfield, 1981; Werner, 2014; Williams & Ridpath, 1982). When the monsoon rain begins, many of the rivers develop extensive floodplains and adult buffalo spread out into the floodplains to drink, wallow and forage in the daytime on the newly emerged and extensive wetland vegetation (Tulloch, 1969; Tulloch & Litchfield, 1981; Werner, 2014). The annual seasonal pulse of inundation, therefore, extends the spatial area of favourable habitat that buffalo readily take advantage of.

Despite these seasonal fluctuations in home range, we found that the core home range area of an individual buffalo was relatively stable, as indicated by the high level of overlap among the three seasonal home range areas for the same buffalo (73% on average). This result agrees with similar findings on buffalo in other parts of the NT that also show that individuals have very high site fidelity (Campbell et al., 2021; Tulloch, 1969). Similar tracking studies of 11 female buffalo in wetland and upland areas of Kakadu also demonstrated that buffalo and their family clan remain faithful to their home range area through seasons and showed no evidence of migration during their study (1 year) (Campbell et al., 2021). Detailed field observational studies by Tulloch (1979, 1969), also reported that buffalo are very reluctant to move out of their home range area. Even during periods where water or food becomes scarce, buffalo remain faithful to the well‐known areas of their home ranges rather than dispersing in search of resources elsewhere (Tulloch, 1969). This is particularly true for females, as any calves they produce remain with them for 2–3 years (Tulloch, 1979) and this is not conducive to migration elsewhere; furthermore, senior males remain with their females until death or dislodged by younger healthy males (when an individual buffalo is observed as alone, it is most often a very young male; Werner, 2014 and references therein).

This high site fidelity is particularly useful from a management perspective as existing buffalo clans and groups are very likely to remain local, making management units easier to discern than for animals with greater dispersal. What is not well known, however, is how the spatial arrangement of buffalo clans and their large and dynamic home ranges is distributed in relation to the local and Indigenous landowners and respective family groups. If neighbouring Indigenous communities have differing management desires for buffalo (e.g. sustainable harvest of buffalo in one community, versus complete removal by another), issues may arise when buffalo clans have large home ranges that span multiple Indigenous communities. Thus, coupling buffalo home range distributions with the spatial configuration of local Indigenous communities could be helpful to delineate buffalo management units to circumvent potential issues.

Our finding that home range crossing time of female buffalo was typically a few days, and particularly shorter for the dry season (a mean of 1.9 days in the dry season vs. 3.2 days for the wet season), is also likely to be reflective of the change in resources between the seasons. During the dry season, buffalo home ranges were smaller, and the shorter home range crossing time also suggests that buffalo are more likely to be revisiting the same sites within their home ranges more often. Campbell et al. (2021) also found greater site revisitations during the dry season for buffalo tracked in Kakadu, NT (although this was relatively stronger for upland savannah buffalo than for floodplain buffalo). On a finer scale, Forrest et al. (2024) also showed that daily buffalo movement often follows cyclical patterns as buffalo move between important habitats such as woodlands and floodplains in the landscape.

These results suggest that removing buffalo during the dry season when home ranges are more compact and with shorter range crossing time presenting more opportunities for optimising buffalo mustering or culling practices. Practitioners needing to search for buffalo in vast landscapes can potentially capitalise on the greater predictability of buffalo movement during this season. For example, mustering operations may search over large distances using helicopters to locate buffalo and identify suitable mustering sites for on ground‐capture and harvesting. Once a dry season survey has been conducted, buffalo tracks can be used to establish suitable remote camps near high‐density areas. We recognise that within our study system, the level of buffalo removal pressure (i.e. hunting, mustering or culling) was unknown and thus could make our results less applicable to other areas with a different buffalo removal pressure. However, previous studies within the NT have also found high site fidelity and predictability in buffalo movement and behaviour, suggesting that these traits are relatively well conserved across different circumstances (Campbell et al., 2021; Tulloch, 1969). Thus, our finding of relatively small daily movements by buffalo, the predictability of home ranges, high site fidelity and buffalo reluctance to leave their home ranges is well supported and indicates that various buffalo removal operations can be optimised with greater confidence.

Our comparison of home range overlap among individual buffalo between the seasonal periods revealed a high level of variation for the propensity of buffalo to share space. This is consistent with field observations of social behaviour and structure. Our research did show that the level of spatial overlap among individual buffalo was generally low during both the wet and dry seasons, which is likely explained by the fact that not only are buffalo strong range residents (and thus less likely to disperse), but they also tend to remain in close‐knit familial clans (Campbell et al., 2021; Tulloch, 1969, 1979). Familial clans are typically made up of a matriarch and her own grown offspring and their calves, while breeding males (other than the dominant male of a family clan) either remain solitary or form separate bachelor herds (Tulloch, 1979). Although buffalo do not typically appear aggressive to other buffalo, the buffalo clans are known to scent mark trees, presumably to communicate their presence to other buffalo in the area and do not tend to mix with other buffalo not in their clan (Tulloch, 1969, 1979). Here, we found that some individual buffalo consistently had very high levels of overlap (approximately 6–8 groupings of buffalo that overlapped 70% or more of their home range area), which is likely to reflect buffalo that belong to the same clan and thus move, feed and wallow together creating substantial home range overlap. From tracking data, low levels of overlap may be indicative of boundaries between neighbouring groups (Noonan et al., 2021).

Interestingly, we also found that overall, in the wet season, buffalo sharing some of their home range area with other buffalo was more frequent (e.g. 30% of different buffalo pairs shared at least some of the home range with other buffalo compared to 10% of buffalo pairs during the dry season). The higher incidences of home range overlap during the wet season may indicate buffalo clans expanding their home ranges when habitat conditions are favourable and tolerating more overlap with neighbouring clans. This is also supported by our finding that the encounter area for the wet season was more than twice as large as the encounter area for the dry seasons, and particularly so for the floodplain area. This greater incidence of potential encounters in the wet season may be driven therefore by the abundance of desirable resources that draw buffalo into common areas and where competition for forage is much less than during the dry season. Further on‐ground research would be useful, however, to determine the exact drivers of these patterns.

### 4.1 Implications for management and future research

Our finding that the likelihood of buffalo sharing resources or interacting is different between wet and dry seasons has important implications for disease management for the region. Feral buffalo are susceptible to several highly contagious diseases that pose a threat to the integrity of the livestock industry, and human health. Brucellosis and tuberculosis are two well‐known examples, but there are others of growing concern (Bradshaw et al., 2012; Freeland & Boulton, 1990; Neverauskas et al., 2015; Rumiano et al., 2021; Stanger & Bowden, 2022). Some of these diseases can be transmitted passively through the environment, such as the bacterial disease leptospirosis that can survive in contaminated soil and water and remain there for several months (Yanagihara et al., 2022). Other pathogens such as lumpy skin disease (LSD, *Capripoxvirus*: Poxviridae), a highly infectious viral disease, can be transmitted through mosquitoes, and thus can be spread even if animals do not come into direct contact (Stanger & Bowden, 2022). Although never detected in Australia, in 2023, LSD was detected in Australian cattle in Indonesia and resulted in a nearly three‐month cessation of live bovine exports. This highlights the importance of disease vigilance, even for diseases never detected in Australia.

Although the Brucellosis and Tuberculosis Eradication Campaign (cf. Introduction) was successful at curbing the risks of disease transmission, buffalo numbers have risen considerably since its cessation in 1997 and these numbers continue to grow (Saalfeld, 2014). The consequent risk of disease outbreak has increased. Here, we have demonstrated that the wet season in particular is likely to create the ideal conditions for the transmission of some of these diseases. This is because buffalo are sharing more space and are more likely to interact with individuals from other clans. The additional free‐standing water is also likely to improve breeding conditions for vectors such as mosquitoes and other biting insects. Taken together, our results suggest that to keep abreast of disease outbreaks, increased disease surveillance efforts may be required during the wet season when transmission risk is high. In addition, the timing of culling practices to occur prior to the wet season could also contribute to minimising transmission risks.

Here, we have generated several important insights for the management of feral buffalo in the NT. However, general inferences should be made with care. Due to the difficult and dangerous nature of tagging buffalo in remote locations, and the challenges of immobilising male buffalo, we tagged only female buffalo. This meant that our results are likely to be more indicative of the spatial and social dynamics of matriarchal buffalo clans. Males may behave differently with regard to the variables that we measured for individual females. Earlier field observations do indicate that male relationships with other buffalo and with family clans are not the same as females (Tulloch, 1969, 1979; Bowman & Corbett, 2023; cf. Introduction above). Bowman and Corbett (2023) indicated that widely dispersed individuals found well outside the typical range of buffalos are males. Tulloch (1969) suggested that groups of young males that are ejected from their birth clan can form new small herds and may behave similarly to other family clans. Adult male buffalo are often the target for mustering practices due to their large size and high market value. This and their potentially differing movement indicate that a future study on male buffalo is warranted. Furthermore, our quantification of individual home range overlap and encounter area of female buffalo, while robust, is limited by sample size and lack of ground truthing, which may warrant further study with a larger number of individuals. A more comprehensive delineation of clans may prove to be a useful management tool in the future to inform disease dynamics research and expedite buffalo harvesting and culling.

This study focussed on floodplain buffalos. Although floodplains are very common across the northern part of NT and are the most common habitat for feral water buffalo, there are other habitat types used by buffalo, for example, the sandstones of central Arnhem land escarpment and in Kakadu, flat uplands savannahs and open grassland downs (Petty et al., 2007). Further research on the behaviour and ecology of buffalo inhabiting other habitat types is required. Here, we have demonstrated for the female floodplain buffalo, patterns of seasonal and social behaviour among buffalo have important management insights that have potential for reducing the negative impacts of feral buffalo. However, our understanding of the broader social and spatial dynamics of buffalo and how these dynamics may change through extreme seasons or differing habitat types remains limited. Answering these questions requires broad‐scale buffalo tagging, and deployment over long periods, especially if decadal variation in weather and climate patterns, and the buffalos' responses, are to be elucidated and translated to support management. Ultimately, for comprehensive management strategies, we recommend future research and planning continue to be in consultation with Indigenous communities that own and manage much of the land that buffalo inhabit. The Indigenous landholders and communities possess extensive ecological understanding, and first‐hand knowledge and experience with buffalo that make their perspectives and management input very valuable for our understanding of buffalo dynamics in the NT.

## AUTHOR CONTRIBUTIONS

**Kyana N. Pike:** Conceptualization (lead); data curation (lead); formal analysis (lead); visualization (lead); writing – original draft (lead); writing – review and editing (lead). **Justin Perry:** Data curation (equal); methodology (equal); validation (equal); writing – review and editing (equal). **Eric Vanderduys:** Data curation (equal); methodology (equal); writing – original draft (equal). **John P. Y. Arnould:** Investigation (equal); validation (equal); writing – original draft (equal). **Andrew Hoskins:** Conceptualization (equal); formal analysis (supporting); methodology (equal); project administration (lead); supervision (equal); writing – review and editing (equal).

## FUNDING INFORMATION

This work was funded by the federal government's Department of Agriculture, Fisheries and Forestry (4‐CU1MYVJ) under the Control tools and technologies for established pest animals and weeds competitive grants programme and the Smart Farming Partnerships Program (round 2).

## CONFLICT OF INTEREST STATEMENT

The authors have no conflicts of interest to declare.

## Supporting information


Appendix S1.

## Data Availability

Data and code associated with this manuscript have been deposited in the public repository Zenodo https://zenodo.org/doi/10.5281/zenodo.11123914.
