# Supplementary material for: Love thy neighbour: Feral buffalos show greater space use, resource overlap and encounters during the wet season in the Northern Territory
Source: Ecol Evol. 2024 Oct 22;14(10):e70345. doi: 10.1002/ece3.70345 (PMC11496382; doi:10.1002/ece3.70345)
Supplement: Supplementary file 1 — Appendix S1. [file ECE3-14-e70345-s001.docx]

**Supplementary material:**

**Love thy neighbour: feral buffalo show greater space use, resource overlap and encounters during the wet season in the Northern Territory**

Kyana N. Pike ^a^[
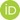
](https://orcid.org/0000-0001-9259-2899), Justin Perry^c^, Eric Vanderduys^d^, John P.Y. Arnould^e^, and Andrew Hoskins ^a,b^

^a^ Health & Biosecurity / Environment, Commonwealth Scientific and Industrial Research Organisation (CSIRO), Townsville, QLD 4811, Australia
^b^ College of Public Health Medical and Veterinary Sciences, Australian Institute of Tropical Health and Medicine, James Cook University, Townsville, QLD 4811, Australia

^c^ Northern Australian Indigenous Land and Sea Management Alliance, NT, Australia

^d^ Environment, CSIRO, Dutton Park, QLD 4102, Australia

^e^ School of Life & Env Sciences, Deakin University, Melbourne, VIC 3125, Australia

**Table S1**. Buffalo dry season home range (2018 only) area estimates for the 95% AKDE, with upper and lower confidence intervals for the estimate. Ne indicates the effective sample size used to estimate home range area for each of the 16 buffalo during the dry season in 2018 in the Djelk region of Northern Territory Australia.

| **Buffalo tag ID** | **Ne** | **Lower CI** | **HR area km2** | **Higher CI** |
| --- | --- | --- | --- | --- |
| 2005 | 15.396 | 112.569 | 199.407 | 310.663 |
| 2014 | 49.535 | 97.782 | 131.941 | 171.138 |
| 2018 | 83.601 | 43.374 | 54.409 | 66.676 |
| 2021 | 80.756 | 25.244 | 31.8 | 39.101 |
| 2022 | 62.972 | 47.469 | 61.777 | 77.943 |
| 2024 | 178.013 | 25.628 | 29.852 | 34.394 |
| 2039 | 131.796 | 24.967 | 29.844 | 35.15 |
| 2043 | 17.316 | 37.259 | 63.601 | 96.872 |
| 2154 | 293.899 | 10.151 | 11.419 | 12.761 |
| 2158 | 9.597 | 225.934 | 479.783 | 827.598 |
| 2223 | 79.999 | 49.736 | 62.723 | 77.195 |
| 2327 | 360.265 | 8.378 | 9.315 | 10.301 |
| 2346 | 25.425 | 11.41 | 17.56 | 25.014 |
| 2354 | 27.924 | 69.76 | 105.047 | 147.441 |
| 2387 | 224.997 | 22.392 | 25.632 | 29.088 |
| 2393 | 252.511 | 30.381 | 34.507 | 38.891 |

**Table S2**. Buffalo dry season home range (2019 only) area estimates for the 95% AKDE, with upper and lower confidence intervals for the estimate. Ne indicates the effective sample size used to estimate home range area for each of the 11 buffalo during the dry season in 2019 in the Djelk region of Northern Territory Australia.

| **Buffalo tag ID** | **Ne** | **Lower CI** | **HR area km2** | **Higher CI** |
| --- | --- | --- | --- | --- |
| 2005 | 37.967 | 55.383 | 78.274 | 105.064 |
| 2014 | 44.833 | 37.716 | 51.74 | 67.947 |
| 2018 | 72.39 | 20.775 | 26.533 | 32.985 |
| 2021 | 88.784 | 2.116 | 2.635 | 3.211 |
| 2022 | 82.677 | 15.626 | 19.628 | 24.079 |
| 2024 | 14.259 | 53.084 | 96.484 | 152.634 |
| 2154 | 326.139 | 4.874 | 5.449 | 6.056 |
| 2158 | 67.937 | 30.001 | 38.639 | 48.353 |
| 2327 | 85.741 | 43.104 | 53.908 | 65.902 |
| 2354 | 158.781 | 19.758 | 23.23 | 26.98 |
| 2387 | 159.967 | 27.779 | 32.642 | 37.89 |

**Table S3**. Buffalo wet season home range area estimates for the 95% AKDE, with upper and lower confidence intervals for the estimate. Ne indicates the effective sample size used to estimate home range area for each of the 16 buffalo during the wet season in 2018—2019 in the Djelk region of Northern Territory Australia.

| **Buffalo tag ID** | **Ne** | **Lower CI** | **HR area km2** | **Higher CI** |
| --- | --- | --- | --- | --- |
| 2005 | 15.396 | 112.569 | 199.407 | 310.663 |
| 2014 | 49.535 | 97.782 | 131.941 | 171.138 |
| 2018 | 83.601 | 43.374 | 54.409 | 66.676 |
| 2021 | 80.756 | 25.244 | 31.8 | 39.101 |
| 2022 | 62.972 | 47.469 | 61.777 | 77.943 |
| 2024 | 178.013 | 25.628 | 29.852 | 34.394 |
| 2039 | 131.796 | 24.967 | 29.844 | 35.15 |
| 2043 | 17.316 | 37.259 | 63.601 | 96.872 |
| 2154 | 293.899 | 10.151 | 11.419 | 12.761 |
| 2158 | 9.597 | 225.934 | 479.783 | 827.598 |
| 2223 | 79.999 | 49.736 | 62.723 | 77.195 |
| 2327 | 360.265 | 8.378 | 9.315 | 10.301 |
| 2346 | 25.425 | 11.41 | 17.56 | 25.014 |
| 2354 | 27.924 | 69.76 | 105.047 | 147.441 |
| 2387 | 224.997 | 22.392 | 25.632 | 29.088 |
| 2393 | 252.511 | 30.381 | 34.507 | 38.891 |

**Table S4**. Proportions of home range overlap for the same buffalo among three consecutive seasons (dry season 2018, wet season 2018 to 2019 and dry season 2019) for the 11 buffalo that had a home range estimate across all seasonal periods. Confidence intervals show the lower and upper intervals for the maximum likelihood estimate.

| **Buffalo tag ID** | **Lower CI** | **Proportion of overlap** | **Higher CI** | **Season 1** | **Season 2** |
| --- | --- | --- | --- | --- | --- |
| 2005 | 0.355 | 0.635 | 0.895 | dry 2018 | dry 2019 |
| 2005 | 0.290 | 0.509 | 0.754 | dry 2018 | wet 2018-2019 |
| 2005 | 0.762 | 0.872 | 0.953 | dry 2019 | wet 2018-2019 |
| 2014 | 0.661 | 0.780 | 0.883 | dry 2018 | dry 2019 |
| 2014 | 0.376 | 0.495 | 0.624 | dry 2018 | wet 2018-2019 |
| 2014 | 0.752 | 0.855 | 0.937 | dry 2019 | wet 2018-2019 |
| 2018 | 0.660 | 0.806 | 0.922 | dry 2018 | dry 2019 |
| 2018 | 0.715 | 0.877 | 0.978 | dry 2018 | wet 2018-2019 |
| 2018 | 0.872 | 0.933 | 0.976 | dry 2019 | wet 2018-2019 |
| 2021 | 0.303 | 0.429 | 0.573 | dry 2018 | dry 2019 |
| 2021 | 0.764 | 0.903 | 0.985 | dry 2018 | wet 2018-2019 |
| 2021 | 0.540 | 0.637 | 0.732 | dry 2019 | wet 2018-2019 |
| 2022 | 0.653 | 0.800 | 0.918 | dry 2018 | dry 2019 |
| 2022 | 0.749 | 0.878 | 0.967 | dry 2018 | wet 2018-2019 |
| 2022 | 0.764 | 0.837 | 0.900 | dry 2019 | wet 2018-2019 |
| 2024 | 0.623 | 0.845 | 0.981 | dry 2018 | dry 2019 |
| 2024 | 0.492 | 0.635 | 0.775 | dry 2018 | wet 2018-2019 |
| 2024 | 0.638 | 0.768 | 0.880 | dry 2019 | wet 2018-2019 |
| 2154 | 0.901 | 0.919 | 0.935 | dry 2018 | dry 2019 |
| 2154 | 0.845 | 0.895 | 0.936 | dry 2018 | wet 2018-2019 |
| 2154 | 0.817 | 0.859 | 0.897 | dry 2019 | wet 2018-2019 |
| 2158 | 0.005 | 0.013 | 0.030 | dry 2018 | dry 2019 |
| 2158 | 0.225 | 0.340 | 0.480 | dry 2018 | wet 2018-2019 |
| 2158 | 0.259 | 0.397 | 0.561 | dry 2019 | wet 2018-2019 |
| 2327 | 0.527 | 0.603 | 0.680 | dry 2018 | dry 2019 |
| 2327 | 0.890 | 0.913 | 0.934 | dry 2018 | wet 2018-2019 |
| 2327 | 0.585 | 0.675 | 0.762 | dry 2019 | wet 2018-2019 |
| 2354 | 0.721 | 0.784 | 0.842 | dry 2018 | dry 2019 |
| 2354 | 0.618 | 0.727 | 0.827 | dry 2018 | wet 2018-2019 |
| 2354 | 0.661 | 0.779 | 0.881 | dry 2019 | wet 2018-2019 |
| 2387 | 0.810 | 0.852 | 0.890 | dry 2018 | dry 2019 |
| 2387 | 0.905 | 0.934 | 0.959 | dry 2018 | wet 2018-2019 |
| 2387 | 0.925 | 0.944 | 0.960 | dry 2019 | wet 2018-2019 |


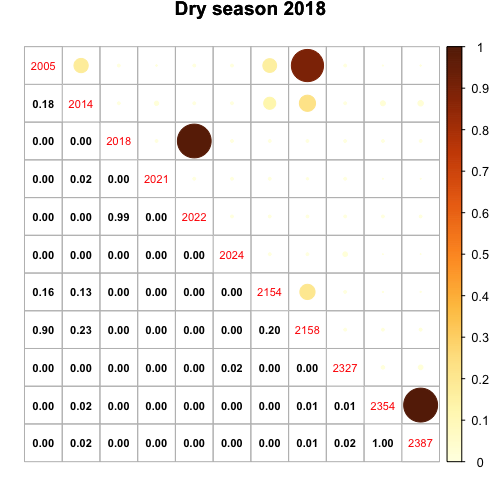


**Figure S1.** Pairwise comparisons of home range overlap among all tagged buffalo in the Djelk area of the Northern Territory, Australia. The central values in red show the tag ID of all 11 buffalo, the lower panel has the numerical proportion of home range overlap between buffalo pairs and the upper panels depicts the overlap values visually, with the larger and darker the circles indicating the higher the home range overlap.
